# Correlation between microneutralization test and a multiplexed immunoassay for evaluation of monkeypox and vaccinia virus antibodies before and after smallpox vaccination

**DOI:** 10.3389/fimmu.2025.1585284

**Published:** 2025-06-23

**Authors:** Serena Marchi, Giulia Piccini, Edmond J. Remarque, Giulia Roscia, Bianca Semplici, Paolo Cantaloni, Noemi Guerrini, Roberta Zannella, Rosa Coluccio, Linda Benincasa, Niccolò Solfanelli, Claudia Maria Trombetta, Emanuele Montomoli, Alessandro Manenti

**Affiliations:** ^1^ Department of Molecular and Developmental Medicine, University of Siena, Siena, Italy; ^2^ VisMederi Srl, Siena, Italy; ^3^ Department of Virology, Biomedical Primate Research Centre, Rijswijk, Netherlands; ^4^ VaepiX, Joint Research Laboratory, University of Siena, Siena, Italy; ^5^ VisMederi Research Srl, Siena, Italy

**Keywords:** monkeypox virus, vaccinia virus, binding and neutralizing antibodies, crossreactive antibody response, humoral immunity

## Abstract

**Introduction:**

Monkeypox (mpox), an endemic zoonotic viral disease in Central and Western Africa, gained international attention in 2022 when clade IIb of the Monkeypox virus (MPXV) spread outside Africa, prompting the World Health Organization (WHO) to declare it a Public Health Emergency of International Concern (PHEIC). Although the PHEIC was lifted in 2023 due to declining global cases, a resurgence caused by clade Ib has reinstated the emergency status. Current mpox vaccines, based on live-attenuated or modified vaccinia virus (VACV), have historical use in smallpox prevention. Understanding the humoral immune response triggered by mpox vaccination and infection, as well as identifying correlates of protection, remain however critical.

**Methods:**

In a previous study, we evaluated the neutralizing antibody response of 1,000 individuals, half born before the cessation of smallpox vaccination in Italy (pre-1975) and half after (post-1979). Higher neutralizing antibody titers against MPXV and VACV were observed in subjects vaccinated against smallpox, indicating a cross-reactive immunity to MPXV. This study further investigated these findings by analyzing the IgG response to five MPXV and five VACV antigens in a subset of the previously tested cohort, using a multiplex immunoassay. Serum samples from 370 individuals were grouped by neutralization profile (negative for both MPXV and VACV, positive for both viruses, negative for MPXV but positive for VACV, and vice versa) and age (born before 1975 and after 1979).

**Results:**

Our data revealed stronger immune responses to specific antigens, particularly A35R/A33R and B6R/B5R, with MPXV-specific binding antibodies showing greater cross-reactivity compared to VACV ones. Furthermore, individuals born before 1975, vaccinated against smallpox, exhibited stronger binding and neutralizing antibody responses, as opposed to people born after 1979 in whom neutralization titers were lower. This suggests that prior VACV-vaccination and subsequent boosting from potential other OPXV encounters in the older population may have resulted in a more VACV-specific immune response over time.

**Discussion:**

This study provides insights into the antigenic determinants of MPXV and VACV antibody cross-reactivity and highlights differences in immune profiles across age and exposure groups. Results obtained suggest that VACV-vaccine imprinting shapes immunity, which could guide the development of more effective vaccine strategies for preventing mpox.

## Introduction

Monkeypox disease (mpox) is a zoonotic viral illness which is endemic in Central and Western Africa. In 2022, the geographic expansion of clade IIb Monkeypox virus (MPXV) outside the African continent has prompted the World Health Organization (WHO) to declare mpox a Public Health Emergency of International Concern (PHEIC) (1). The PHEIC was lifted in 2023 as global cases dropped substantially. However, the emergency status has been recently re-instated due to the multi-country outbreak caused by another MPXV strain, clade Ib (2). According to WHO, from January 2022 to 30 November 2024, 117,663 confirmed cases of mpox have been reported in 127 countries worldwide (3).

Vaccination of high-risk groups is the best strategy to prevent mpox and reduce the severity of disease symptoms. Currently licensed vaccines for prevention of mpox are based on live-attenuated or modified vaccinia virus (VACV) and have been used in the past to fight smallpox. Evidence from surveillance programs conducted in the early 1980s suggest that prior VACV vaccination could be up to 85% effective against mpox (4).

The ability of antibodies raised against VACV to provide cross-protection against MPXV lies in the fact that the viral core region of these two double-stranded DNA Orthopoxviruses (OPXV) exhibit over 95% sequence homology (5). Given the high conservation of OPXV genome, antibody cross-reactivity has been observed even towards other members of the genus such as Cowpox virus (CPXV) (6).

Herd immunity to MPXV and other OPXVs has however waned over time, as routine VACV immunization was discontinued in the late 1980s due to the eradication of smallpox disease (4). As a consequence, the fraction of OPXV-naïve subjects in the population has increased over time.

Understanding the humoral immunity triggered by MPXV infection and VACV vaccination is pivotal to pinpoint antigens that may act as candidates for new vaccines as well as to identify a correlate of protection, which is still missing.

MPXV exists in two antigenically distinct infectious forms: the intracellular mature virion (IMV) and the extracellular enveloped virion (EEV). The surface membranes of these viral forms are characterized by approximately 25 proteins on the IMV and 6 surface proteins on the EEV (7, 8), many of which play crucial roles in viral entry and transmission.

During inter-host transmission, the IMV form predominates, expressing about 25 membrane-associated proteins, including MPXV A29L and E8L proteins. In contrast, the EEV form is primarily involved in intra-host transmission and carries six envelope proteins, including MPXV A35R and M1R proteins (9–11). Six MPXV surface proteins were previously reported to induce antibodies with neutralization capacity and hence with potential as vaccine antigens (12, 13): H3L, E8L, M1R, A29L, A35R and B6R. The VACV orthologs of these viral antigens are, respectively, H3L, D8L, L1R, A27L, A33R and B5R (6, 12, 13).

Among these, MPXV A29L, the ortholog of VACV A27L, plays an essential role in viral replication and entry by binding to heparan sulphate (14–17), while E8L, ortholog of D8L, mediates cell adhesion through binding with chondroitin sulphate (18, 19). MPXV M1R/VACV L1R contributes to IMV assembly and entry (20, 21).

A35R, ortholog of VACV A33R, is an envelope glycoprotein of EEV, that facilitates the effective cell-to-cell spread of viral particles (22, 23), while MPXV B6R/VACV B5R supports viral dissemination and regulates the complement system of the host cell (21). B5R was also found to contribute to the formation of EEV during the wrapping steps of IMV (24, 25).

In a previous study, we investigated the neutralizing antibody response towards MPXV and VACV in a population of 1,000 subjects, half born before the discontinuation of smallpox vaccination, and half after (26). An association between VACV and MPXV antibody levels was observed, corroborating the evidence that VACV-based smallpox vaccines may also confer some degree of cross-protection that can neutralize MPXV infection. However, we observed that this cross-reactivity may not be completely bi-directional.

Interestingly, a small percentage (15.6%) of individuals born after the interruption of anti-smallpox immunization showed positive VACV and/or MPXV neutralization titres (26), suggesting that exposure to similar OPXVs over time may have influenced the observed data.

The aim of the present study is to evaluate the binding antibody response directed towards 5 MPXV antigens (E8L, M1R, A29L, A35R and B6R) and their VACV orthologs (D8L, L1R, A27, A33R and B5R) in a subset of the above-mentioned cohort, via a multiplex immunoassay (MSD).

Correlations between the IgG levels against the 10 antigens and the neutralization titres may help elucidating the antigenic determinants responsible for the cross-reactivity observed in the previously obtained neutralization data. Additionally, although recent research has shown that it is unlikely to distinguish VACV-immunized and MPXV infected individuals using these 10 antigens (27, 28), the MSD data can help understand if there is a trend for increased binding to certain antigens based on the exposure group.

## Materials and methods

### Serum samples

A total of 370 human serum samples were selected from a previous sero-epidemiological study (26). In this previous study, the neutralizing antibody response towards MPXV and VACV was investigated in a population of 1,000 subjects, half born before the discontinuation of smallpox vaccination (29), and half after. These samples were anonymously collected in 2022 in the Apulia region (Southern Italy) as residual samples for unknown diagnostic purposes. For each sample, only the date of collection and the subject’s age and sex were recorded. All serum samples were tested by the established microneutralization cytopathic effect-based assay (hereafter referred to as Virus Neutralization, VN) against MPXV and VACV as previously reported (30).

For this study, the selected 370 serum samples were grouped in 4 exposure groups based on their neutralization profile, i.e., negative for both MPXV and VACV neutralizing antibodies (MPXV- VACV-, hereafter referred to as V-M-), positive for both VACV and MPXV neutralization antibodies (MPXV+ VACV+, or V+M+), negative for MPXV antibodies but positive for VACV ones (MPXV- VACV+, or V+M-) and vice versa (MPXV+ VACV-, or V-M+) (**Table 1**). Samples belonging to the VACV- MPXV- group were randomly selected from all VACV and MPXV VN negative samples from the born after 1979- and the born before 1975- birth cohorts, while the samples in the remaining exposure groups are all available samples fulfilling the specified criteria.

**Table 1:** Serum samples selected from the previous study (26) and grouped according to their neutralization profile (exposure group) and birth cohort.

Exposure group	Abbreviation	Born after 1979	Born before 1975	Total
VACV- MPXV-	V-M-	50	50	100
VACV+ MPXV-	V+M-	44	85	129
VACV- MPXV+	V-M+	19	21	40
VACV+ MPXV+	V+M+	15	86	101
Total		128	242	370

VACV, Vaccinia virus; MPXV, Monkeypox virus.

### MSD assay

Meso Scale Diagnostics (MSD, LLC., Rockville, MD) is an immunoassay technology that use an electrochemiluminescent technology and micro plates with carbon electrodes integrated into each well. The V-PLEX Orthopoxvirus Serology Kit (MSD, LLC., Rockville, MD) quantitatively measures antibodies direct to MPXV and VACV viral proteins. Plates are provided with 10 viral antigens (5 MPXV proteins and their 5 orthologous VACV proteins) as listed in **Table 2**.

**Table 2:** List of antigens detected by the MSD kit: orthologous pairs, antigens description, protein length (number (N) of amino acids (aa), number of differing amino acids, percentage (%) of protein similarity and GenBank references.

Orthologous Pairs	Antigens	Protein length (N aa)	Differing aa (N)	Protein similarity (%)	GenBank Protein	GenBank Nucleotide
VACV A27L/MPXV A29L	VACV A27L	110	6	94.55	ABD52635	DQ121394.1
	MPXV A29L	110			URK20577	ON563414.3
VACV A33R/MPXV A35R	VACV A33R	181	12	93.37	ABD52644	DQ121394.1
	MPXV A35R	181			URK20584	ON563414.3
VACV B5R/MPXV B6R	VACV B5R	317	11	96.53	ABD52686	DQ121394.1
	MPXV B6R	317			URK20605	ON563414.3
VACV D8L/MPXV E8L	VACV D8L	304	17	94.41	ABD52586	DQ121394.1
	MPXV E8L	304			URK20542	ON563414.3
VACV L1R/MPXV M1R	VACV L1R	250	3	98.8	ABD52554	DQ121394.1
	MPXV M1R	250			URK20517	ON563414.3

VACV, Vaccinia virus; MPXV, Monkeypox virus.

MSD assay was performed following the manufacturer’s instructions. Briefly, calibrators and controls were diluted according to the data sheet provided within the kit. Serum samples were diluted 1:100 in Diluent 100, included in the kit. After 30 minutes in blocking solution (Bovine serum albumin in a PBS-based buffer optimized for use with MSD) at room temperature (RT) with shaking, the plates were washed 3 times with 150 µl/well of the wash solution provided; 50 µl of each calibrator, control and diluted serum sample was then added to the plates, which were incubated for 2 hours at RT with shaking. After another washing step, 50 µl well of SULFO-TAG anti-human IgG antibody was added, and the plates were incubated for 1 hour at RT with shaking. After a washing step, 150 µl/well of MSD GOLD Read Buffer B was added, and the plates were read on the MSD instrument.

Calibration curves used to calculate antibody concentrations are established by fitting the signals from the calibrators to a 4-parameter logistic (or sigmoidal dose-response) model with a 1/Y2 weighting. Antibody unit concentrations in controls and diluted samples are determined from their electro chemiluminescent signals by backfitting to the calibration curve.

### Statistical methods

The antibody concentration (AU/ml) for each pox antigen was calculated for each sample and used for the analysis. All statistical analyses were performed with R studio and R version 4.4.2 (31). For each antigen, median antibody levels by birth cohort and exposure group were calculated along with their corresponding interquartile ranges (IQR). Statistical significance was evaluated by Kruskal Wallis test in the four exposure groups (V-M-, V-M+, V+M- and V+M+); if significant a Dunn test was performed for comparisons with Holm’s correction for multiple tests. A Mann Whitney test was used to test equality for the age groups within the four exposure groups. Statistical significance was set at p<0.05, two-tailed. Correlations between VN titres for MPXV and VACV and median antibody levels of the 10 OPXV antigens for exposure groups and for the two birth cohorts was determined by Pearson’s product-moment correlation coefficient (r). Figures were generated using the ggplot2 R and GGally packages.

## Results

### Antibody levels and correlation with age

Binding antibodies towards the 10 OPXV antigens were assessed by MSD in serum samples from the entire study group (N = 370).

The highest antibody levels were observed in subjects born before 1975 (older group), with anti-VACV A33R (median: 27,086; IQR 10,949 – 81,695 (V-M+)), anti-MPXV A35R (median: 19,795; IQR 6,372 – 52,485 (V+M+)), anti-VACV B5R (median: 22,333; IQR 4,461 – 58,064 (V+M+)) and anti-MPXV B6R (median: 20,780; IQR 4,516 – 53,952 (V+M+)) antibodies being the most abundant (**Supplementary Table S1**).

**Figure 1:**
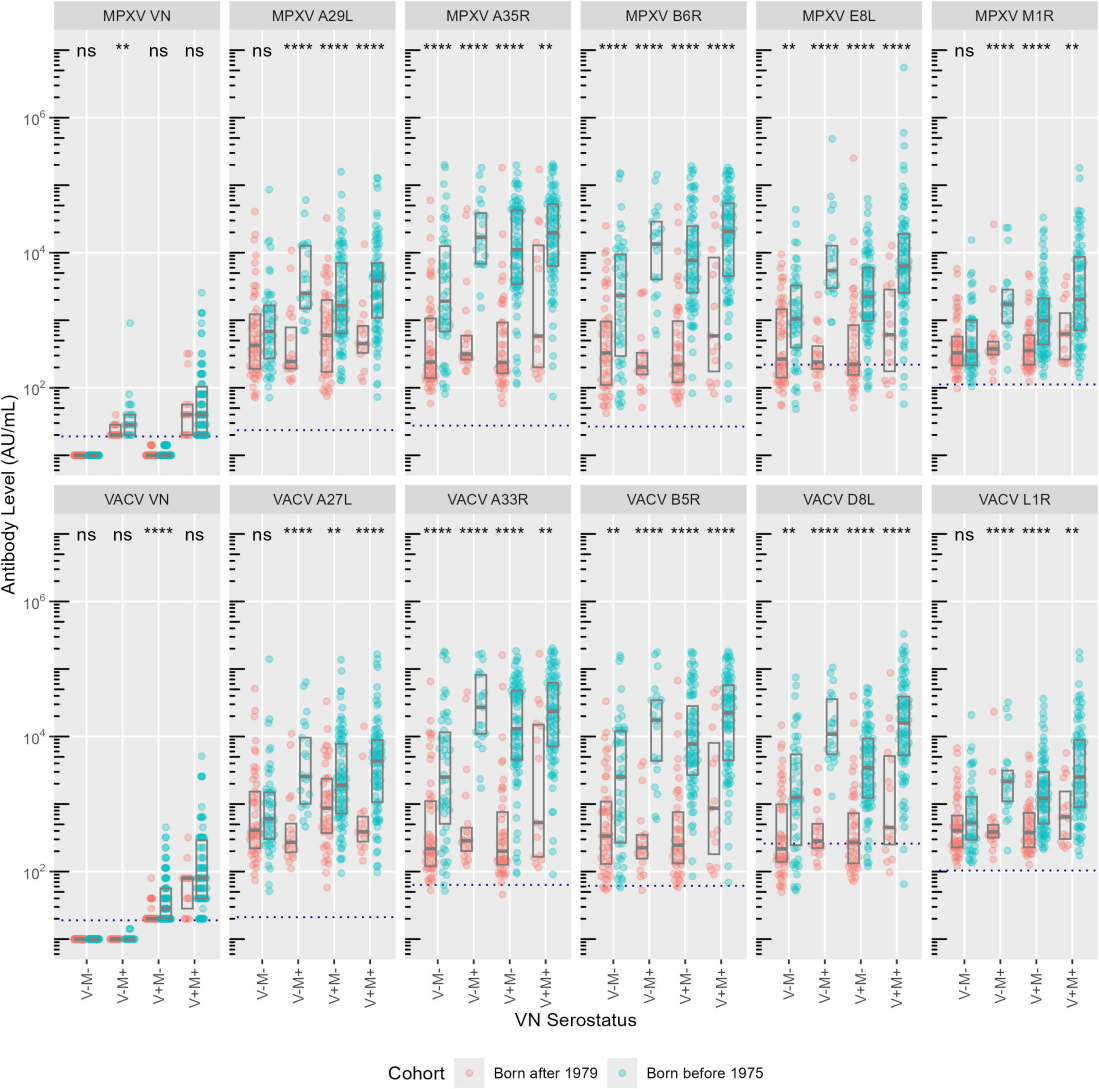
Antibody levels (AU/ml) per monkeypox (MPXV, upper panels) and vaccinia (VACV, lower panels) antigens. Exposure groups (VN serostatus) are reported on the x-axis, antibody levels for each subject are reported on the y-axis. Colors indicate birth cohort (red: born after 1979; blue: born before 1975). Boxes represent median and interquartile range. Dashed lines indicate cut-off values as defined by Hicks et al. (10). Significances from Mann-Whitney U test are indicated: ns = not significant, ** P < 0.01, **** P < 0.0001.

**Figure 2:**
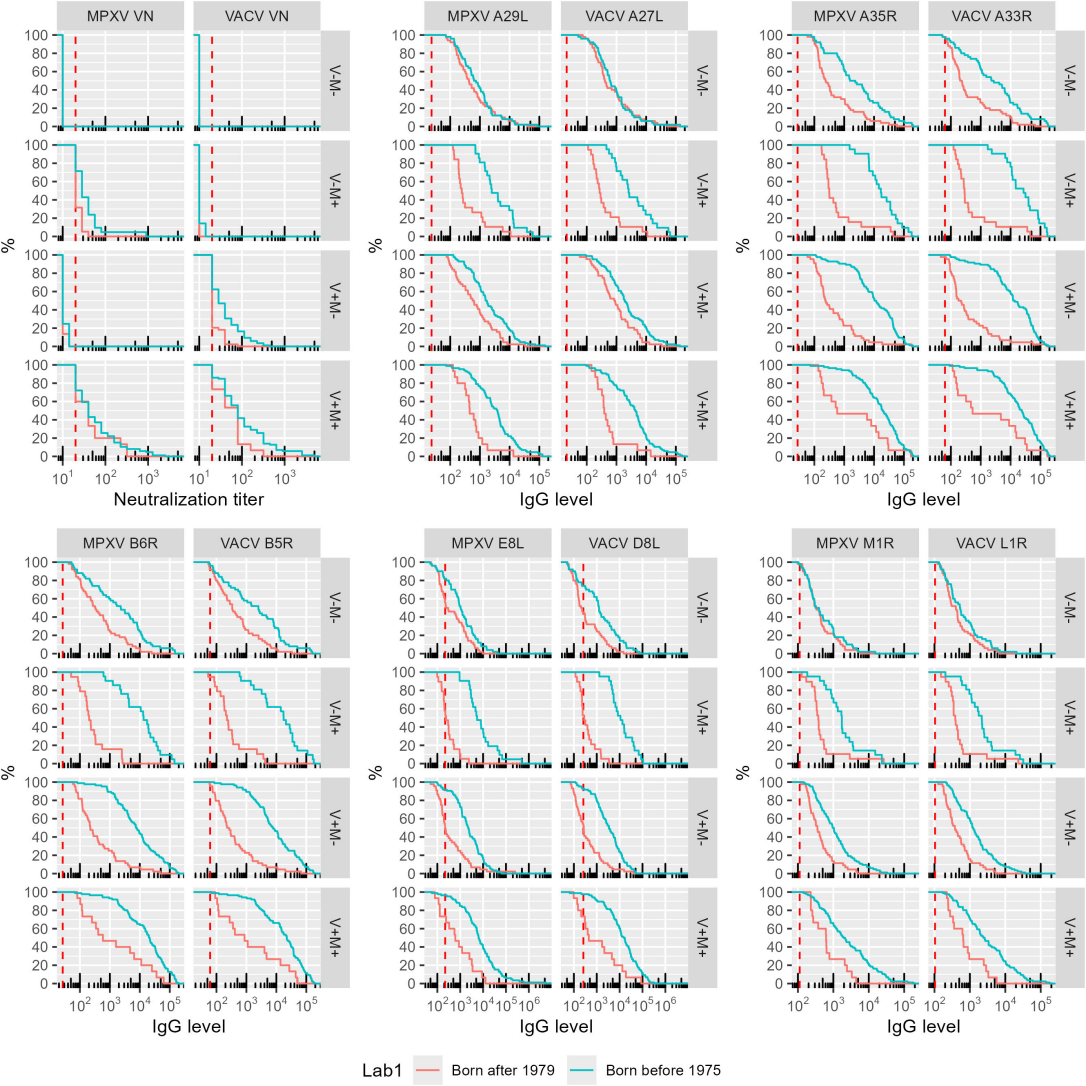
Reverse cumulative distribution of monkeypox (MPXV) and vaccinia (VACV) antigens by orthologs pair. The data are reported as percentage of subjects within each birth cohort and exposure group (VN serostatus) versus assay antibody level. The positivity cut-off value is indicated per assay as the vertical dashed red line. Colors indicate birth cohort (Red: born after 1979 cohort; blue: born before 1975 cohort).

VACV and MPXV VN positive individuals born after 1979 (younger group) displayed significantly lower antibody levels for most of the 10 OPXV antigens (**Figure 1**) as compared to the subjects born before 1975 (older group). In the older age group, antibodies that bind MPXV M1R and VACV L1R were present at lower levels than antibodies binding the other assessed antigens; whereas in the younger cohort, the levels of antibodies towards M1R and L1R were comparable to those directed against the other 8 OPXV antigens (**Figure 1**). Reverse cumulative distribution curves of MPXV and VACV antibody levels for the 10 OPXV antigens (**Figure 2**) confirmed a shift toward higher binding antibody levels in the cohort of those born before 1975.

As the older age group putatively received the smallpox vaccination, which shown to provide some degree of cross-protection against mpox (4), it is not surprising that binding antibody levels in this cohort were significantly lower in negative subjects than in those positive to at least one virus (**Supplementary Figure S1**). Interestingly, however, median antibody concentrations to the 10 OPXV antigens within the older cohort were lower in the V+M- than in the V-M+ group (with the exception of antibodies against the VACV A27L antigen).

Among the 5 orthologue pairs, the antibody response directed against the E8L/D8L in the older group showed the greatest variability with respect to exposure to MPXV or VACV. Antibodies directed to VACV D8L were significantly higher in V-M+ individuals than in V+M- ones (p=0.002). This suggests that older MPXV-positive individuals negative for VACV have antibodies that cross-react with the VACV D8L, but older VACV-positive subjects negative for MPXV do not show the same level of antibody cross-reactivity towards the MPXV antigen E8L. No significant differences among exposure groups were instead observed in the younger cohort (**Supplementary Figure S1**).

The hypothesized disparity in cross-reactive potential highlighted in the older study population is also evident when considering the E8L/D8L antibody ratio according to birth cohort and exposure group (**Supplementary Figure S2**); in each serostatus in which positivity to at least one of the viruses is present, the ratio is always in favour of VACV D8L antigen, including in subjects positive for MPXV antibodies but negative for VACV ones. The antibody response directed towards the orthologue pair A35R/A33R, by contrast, appears to be the one for which the ratio is most consistent with the presumed exposure to MPXV (in the younger group) and VACV (in the older group), with differences that are always significant between the two age groups (V-M-, p = 0.019; V-M+, p < 0.0001; V+M-, p < 0.0001 and V+M+, p = 0.001) (**Supplementary Figure S2**).

In our previous study (26), 44 individuals born after 1979 had positive VACV VN titres despite the lack of positive MPXV VN titres. In addition to the imbalance in the E8L/D8L pair, this observation seems to be also related to the A29L/A27L ortholog pair, for which the V+M- exposure group of this birth cohort shows an imbalance in favour of the VACV A27L antigen (p = 0.03 compared to the birth cohort born before 1975) (**Supplementary Figure S2**). Similarly, 21 individuals in the cohort of those born before 1975 had MPXV positive VN titres but were VACV VN negative. In this case it can be observed that certain orthologous pairs (e.g. A29L/A27L and B6R/B5R) show no significant differences between the M+V- individuals belonging to the two birth cohorts (**Supplementary Figure S2**).

The correlation between binding antibody levels to the 10 OPXV antigens and age in the younger VACV-naïve group seems to increase over time (**Supplementary Figure S3**), contrary to what happens in the older age group (**Supplementary Figure S4**). MPXV and VACV VN titres, instead, seem to increase with age in both age cohorts, with particular reference to the VACV VN titres in the older age group (**Supplementary Figure S4**). While the correlation between neutralizing antibodies and age is always positive (regardless of the birth cohort and virus), the correlation between binding antibodies and age is always positive only in the younger individuals.

### Correlation between binding antibody levels and neutralizing antibody titres

**Figure 3:**
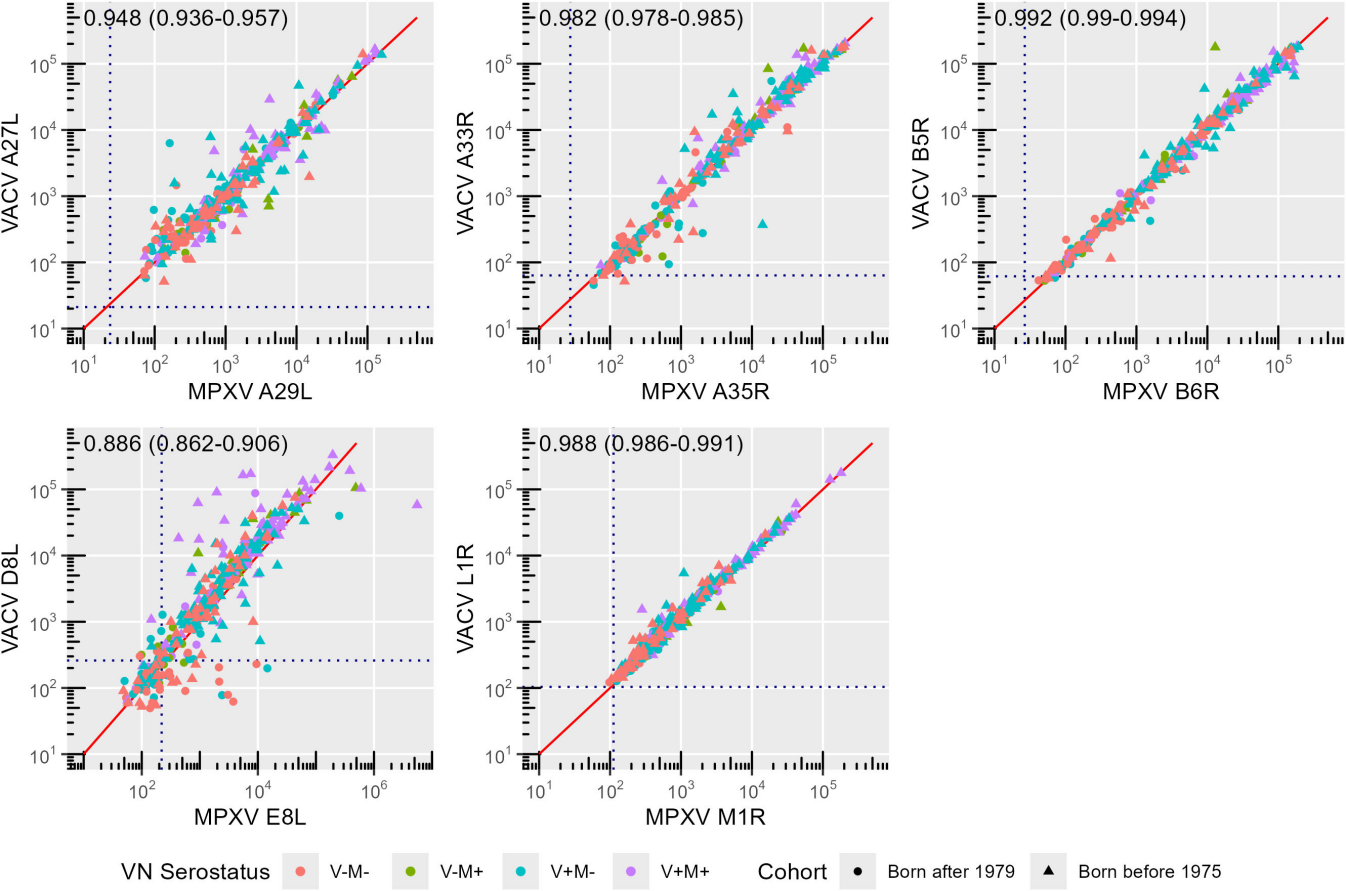
Correlation between monkeypox (MPXV) and Vaccinia (VACV) orthologues. Symbols and colors indicate birth cohort (circle: born after 1979; triangle: born before 1975) and exposure group (VN serostatus; red: V-M-, green: V-M+, blue: V+M-, purple: V+M+), respectively. The solid red line indicates the x = y line. Correlation coefficients along with their 95% confidence interval (CI) are reported for each panel. Dashed lines indicate cut-off values as defined by Hicks et al. (10).

Binding antibody responses to the assessed MPXV and VACV orthologues are strongly correlated (**Figure 3**), reflecting the high sequence homology reported for these antigens pairs (**Table 2**).

The strongest correlation was observed between antibodies targeting the orthologue pair B6R and B5R (r = 0.992) (sequence homology: 96.53%), followed by that between L1R and M1R antibodies (r = 0.988) (sequence homology: 98.80%), A35R and A33R antibodies (r = 0.982) (sequence homology: 93.37%) and A27L and A29L antibodies (r = 0.948) (sequence homology: 94.55%) (**Figure 3**).

**Figure 4:**
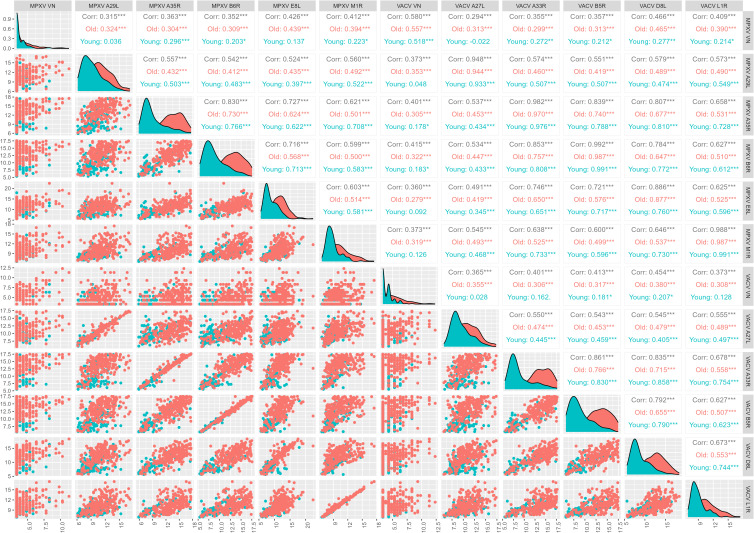
Correlation between monkeypox (MPXV) and Vaccinia (VACV) antigens and virus neutralization (VN) assay (log2-tranformed axes). Total correlations between VN titers for MPXV and VACV and median antibody levels of the 10 OPXV antigens and by birth cohort (born before 1975, old and born after 1979, young) was determined by Pearson’s product-moment correlation coefficient (r).

A robust correlation (r = 0.886) is also shown between anti-E8L and anti-D8L antibodies. However, this is lower than in the other pairs of orthologues (**Figure 3**), despite similarly high sequence homology of E8L and D8L (94.41%) (**Table 2**). The reduced correlation is due to the younger age group (r = 0.760, 95% confidence interval (CI) 0.676 – 0.825) as compared to the older one (r = 0.877, 95% CI 0.844 – 0.903; p = 0.00009) (**Figure 4**). Notably, several non-orthologous antigen pairs also exhibited strong correlations in antibody responses. Analysis of correlations across the full study population revealed that antibodies targeting MPXV A35R were strongly associated with those directed to VACV B5R (r = 0.839), MPXV B6R (r = 0.830), VACV D8L (r = 0.807) and MPXV E8L (r = 0.727). Similarly, the presence of VACV A33R antibodies was highly correlated with that of antibodies against VACV B5R (r = 0.861), MPXV B6R (r = 0.853), VACV D8L (r = 0.835) and MPXV E8L (r = 0.746). A marked correlation has also been observed between MPXV E8L antibodies and those towards MPXV B6R (r = 0.716) and VACV B5R (r = 0.721); as well as between VACV D8L antibodies with those directed to VACV B5R (r = 0.792) and MPXV B6R (r = 0.784) (**Figure 4**). This seems to indicate that exposure to immunodominant antigens such as A35R, A33R, B5R or B6R does not hamper development of immune responses towards less immunogenic targets such as E8L.

**Figure 5:**
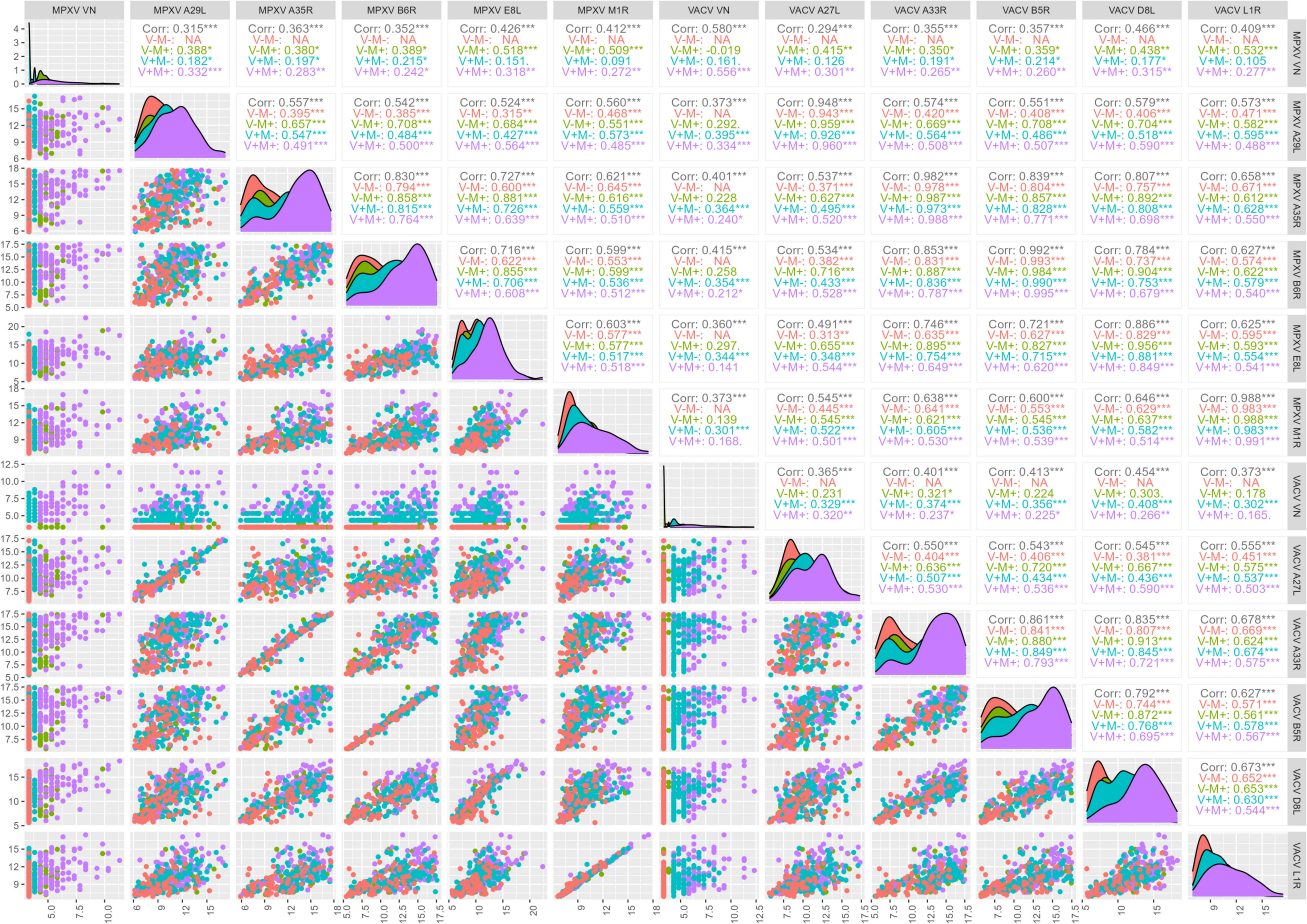
Correlation between monkeypox (MPXV) and Vaccinia (VACV) antigens and virus neutralization (VN) assay (log2-tranformed axes). Total correlations between VN titers for MPXV and VACV and median antibody levels of the 10 OPXV antigens and by exposure group (VACV+ MPXV+, V+M+; VACV+ MPXV-, V+M-; VACV- MPXV+, V-M+; VACV- MPXV-, V-M-) was determined by Pearson’s product-moment correlation coefficient (r).

When analyzing the contribution of age, in 4 out of 5 orthologues pairs (i.e., all except A27L vs A29L) and in most non-orthologue pairs, correlations between VACV- and MPXV- binding antibodies are higher in the younger age group (**Figure 4**). Looking at either orthologues or non-orthologues correlations in the different exposure groups, the highest correlations between binding antibodies are generally in the V-M+ group (**Figure 5**).

The correlation between VACV and MPXV VN titers in the sub-cohort of 370 subjects included the present study is positive but moderate (r = 0.580) and slightly higher in the older age group than in the younger one (Born before 1975, older group: r = 0.557; Born after 1979, younger group: r = 0.518) (**Figure 4**), in line with what previously observed within the larger study group of 1000 subjects (26). This association is entirely due to the V+M+ exposure group (r = 0.556), as extremely low or negative correlations were reported in the V+M- (r = 0.161) and V-M+ (r = -0.019) profiles (**Figure 5**).

Interestingly, both VACV VN and MPXV VN correlate poorly with the 10 OPXV antigens, and some degree of low but significant correlation is nearly entirely due to antibodies in the older age group (**Figure 4**). The only case where a significant, albeit low, correlation was observed between VN and OPXV antigens is the case of MPXV neutralizing antibodies and A35R-binding antibodies in the younger age group (**Figure 4**).

If looking at exposure profiles, the strongest correlations between MPXV VN titers and binding antibodies are observed in the V-M+ groups. In this latter population, MPXV VN shows the highest and most significant associations with MPXV E8L (r = 0.518), MPXV M1R (r = 0.509) and VACV L1R (r = 0.532) antibodies. VACV VN titers exhibited slightly lower (yet statistically significant) and similar correlations with binding antibodies against VACV and MPXV antigens, largely driven by individuals with a V+M- serostatus. The highest association in this case was found with VACV D8L antibodies (r = 0.408) (**Figure 5**).

## Discussion

This study provides an in-depth analysis of binding antibody responses to 10 OPXV antigens, with a particular focus on differences in immune profiles between individuals according to their neutralization activity to MPXV and VACV and their age.

Our data confirm that certain MPXV and VACV antigens elicit stronger immune responses than others. The highest antibody levels were observed for the orthologous pairs A35R/A33R and B6R/B5R, supporting their role as highly immunogenic targets. This is consistent with previous studies indicating that surface proteins, particularly those associated with the EEV form (i.e., A33R/A35R and B5R/B6R), are the most immunodominant (12, 32, 33). In contrast, L1R, although immunogenic, is part of the inner capsid of the IMV and may not be as readily exposed to the immune system in a natural infection context (32). In agreement with previous serological studies (6, 33), the L1R/M1R pair exhibited the lowest antibody levels across all exposure groups. One of the main findings of the present study is that MPXV-specific antibodies exhibit greater binding cross-reactivity and may have potential for higher cross-neutralizing capacity compared to the VACV antibodies. The data suggest in fact that individuals with MPXV-neutralizing titres have stronger binding antibody levels to VACV antigens than those with VACV-neutralizing titers do to MPXV antigens. This is particularly true for antibodies targeting the E8L/D8L orthologue pair, which showed higher binding cross-reactivity in MPXV-positive individuals compared to VACV-positive individuals (**Figure 1** and **Figure 4**). Our analysis also revealed that MPXV E8L-specific antibodies exhibit one of the strongest associations with neutralization activity in MPXV-positive individuals. These findings align with the fact that cross-protection afforded by the two viruses might be asymmetrical, as speculated previously when looking at neutralization data (26). Additionally, the likelihood of being VACV-positive if MPXV-positive and vice versa is lower in the younger age group as compared to the older one. This may once again indicate a non-bi-directional cross-reactivity of the humoral response elicited towards E8L and D8L (**Figure 4**).

Notably, MPXV antibodies in younger individuals appear less functionally mature (i.e., binding but not neutralizing), suggesting that natural infections alone may not elicit a robust neutralizing response. As younger individuals have not been vaccinated against smallpox, they may only have been exposed to MPXV or similar viruses (e.g. CPXV). It is conceivable that young people may only have more specific antibodies to MPXV than to VACV, appearing to be the result of a “primary” response, with lower antibody levels (**Figure 1**) and less specific, i.e., more reactive in terms of binding towards VACV (**Figure 4**). Consequently, it can be hypothesized that the binding response driven by MPXV-specific antibodies is more cross-reactive and broader, and more adept at binding VACV than vice versa.

In older subjects, the imprinting of the historical smallpox vaccination may have exerted a significant influence, with both stronger neutralization and binding responses. The group born before 1975 exhibited considerably higher antibody levels in comparison to the younger age group, irrespective of the antigen considered (**Figure 1**). It is plausible that the older population has received a natural boost over time due to exposure to either smallpox/VACV vaccine boosters (prior to disease eradication) or other OPXV including MPXV. This suggests that prior VACV vaccination followed by subsequent antigenic boosting from potential exposure to other OPXV (including MPXV) has led to the development of a more VACV-specific immune response over time, likely due to vaccine imprinting, rather than a response directed towards MPXV or other OPXV. In our previous study (26), a subset of individuals born after 1979 - who are not supposed to have been exposed to VACV due to the cessation of smallpox vaccination in the 1980s - exhibited positive VACV neutralization titres despite lacking MPXV-neutralizing activity. We hypothesize that this could be explained by the observed imbalance in the immune response to the E8L/D8L antigen pair, potentially leading to antibodies that preferentially cross-react with the VACV D8L antigen, regardless of whether exposure was to MPXV or VACV. In addition, it may also be related to the differential cross-reactivity towards the A29L/A27L ortholog pair: among people born after 1979, those belonging to the V+M- exposure group have an imbalance in the antibody ratio in favour of the VACV A27L antigen compared to the MPXV A29 antigen (**Supplementary Figure S2**). This raised the possibility that exposure to other OPXV, such as CPXV or other zoonotic poxviruses (34, 35), may have contributed to the observed data, as also reported in other serological studies (36, 37). Similarly, some individuals in the cohort of those born before 1975 displayed MPXV-neutralizing antibodies but lacked neutralizing activity against VACV. This not expected as this population has likely been immunized with VACV via the smallpox vaccine. It can be however observed that the magnitude of the humoral response directed to certain orthologous pairs (such as A29L/A27L and B6R/B5R) is similar between the V-M+ individuals across to the two birth cohorts, as shown by the absence of statistically significant differences in antibody ratio (**Supplementary Figure S2**), indicating that antibodies elicited upon exposure to certain antigens may have the same capability to bind the corresponding orthologue and further emphasizing the complexity of cross-reactive immune responses in different age cohorts. Overall, results of the present study in the two sub-groups evaluated showed that antibodies in the young unvaccinated population (who has likely fewer chances of encountering OPXV antigens with respect to older individuals) tend to increase their binding rather than their neutralizing capacity (**Supplementary Figure S3**), as opposed to the older population (**Supplementary Figure S4**). The humoral response in this latter case may be the result of both VACV vaccine imprinting and possible natural boosters from CPXV or other zoonotic poxviruses infections (hybrid immunity), which may have induced mainly VACV-reactive antibodies with increased specificity and neutralizing capacity for VACV.

Surprisingly, despite the high correlation observed between binding humoral responses towards MPXV and VACV orthologous antigen pairs (**Figure 3**), the association between MPXV and VACV neutralizing antibodies remains moderate regardless of the age group (**Figure 4**), and entirely attributed to the presence of antibodies against both viruses (V+M+ serostatus) (**Figure 5**). This could mean that MPXV antibodies alone (V-M+ exposure group), which are presumably not sufficiently boosted and resulting only from (limited) natural infections, are cross-reactive and broader in their binding ability but not in their neutralizing ability. This is particularly evident in the younger population, in which antibodies displayed an enhanced capability for binding over neutralization (**Supplementary Figure S3**). Similarly, the presence of VACV antibodies alone (V+M- profile) may not be adequate to provide adequate cross-neutralization of MPXV, especially considering that potential exposures to other OPXV over time may lead to a more VACV-specific neutralizing response in smallpox-vaccinated subjects, as observed in the older population of this study (**Supplementary Figure S4**). Indeed, VACV and MPXV neutralizing titers correlate better with antibodies that bind the homologous virus, as shown by the fact that the highest associations are observed in the V+M- group for VACV neutralizing antibodies and in the V-M+ group for MPXV neutralizing antibodies (**Figure 5**). Collectively, these findings underscore the importance of repeated immunizations with MPXV antigens to enhance the development of cross-neutralizing antibodies against MPXV.

Our study has some limitations. Firstly, there is missing information on smallpox vaccination of the subjects as well as their clinical or travel history. This includes information on previous MPXV or other OPXV infections. Other factors, such as underlying health conditions (e.g. autoimmune disorders or immunocompromising conditions), may also influence immunological responses (38, 39). We have used the cut-off values as reported by Hicks et al. (27) as a reference, but these are only partially helpful in distiguishing between the different exposure groups from our study. It would have been useful to determine what the cut-off values would be for the 10 antigens in non-OPXV exposed subjects, which we are unable to do due to the unavailability of samples and tests. Given the recent MPXV outbreak, this study is especially pertinent. It explores how prior smallpox vaccination or OPXV exposure affects the immune response to the current MPXV strain, thereby examining cross-protective immunity between MPXV and other OPXV, including smallpox. The observed differences in immune responses between younger and older individuals highlight the impact of historical smallpox vaccination, suggesting that vaccine imprinting plays a crucial role in shaping long-term immunity against OPXV, including MPXV. The development of antibody responses over time, as well as their affinity, avidity, and cross-reactivity, may be better understood through a longitudinal study. Future studies should explore whether modern vaccines can be optimized to induce similarly robust responses in younger populations who lack prior smallpox vaccination-induced immunity.

In conclusion, this study investigated the antigenic determinants of previously observed broadly neutralizing antibody responses, to elucidate the actual breadth of serum cross-neutralization to VACV and MPXV. These findings contribute to our understanding of the differential immune responses to MPXV based on vaccination history and age-related exposure and provide valuable insights for the development of next-generation MPXV vaccines.

## Data Availability

The original contributions presented in the study are included in the article/**Supplementary Material**, further inquiries can be directed to the corresponding author/s.

## Supplementary material

Supplementary Figure 1 Antibody levels (AU/ml) per monkeypox (MPXV, left panels) and vaccinia (VACV, right panels) antigens per birth cohort (born after 1979, upper panels; born after 1975, lower panels). Exposure groups (VN serostatus) are reported on the x-axis, antibody levels for each subject are reported on the y-axis. Boxes represent median and interquartile range. Dashed lines indicate cut-off values as defined by Hicks et al. (10). Significances from Kruskal-Wallis test followed by Dunn test are indicated: ns, not significant, *P < 0.05, **P < 0.01, ***P < 0.001. ****P < 0.0001.

Supplementary Figure 2 Ratio Monkeypox (MPXV)/Vaccinia (VACV) for each ortholog pair by exposure group and birth cohort. Exposure groups (VN serostatus) are reported on the x-axis, MPXV/VACV ratio for each subject is reported on the y-axis. Colors indicate birth cohort (red: born after 1979; blue: born before 1975). Boxes represent median and interquartile range. Significances from Mann-Whitney U test are indicated: ns, not significant, *P < 0.05, **P < 0.01, ***P < 0.001. ****P < 0.0001.

Supplementary Figure 3 Correlation between antibody levels and age for the born after 1979 cohort. The subjects in ascending order of age are reported on the x-axis, individual antibody level for each assay/antigen is reported on the y-axis. Dashed lines indicate cut-off values as defined by Hicks et al. (10). The solid red line indicates linear least squares regression line and gray area indicates the 95% confidence interval.

Supplementary Figure 4 Correlation between antibody levels and age for the born before 1975 cohort. The subjects in ascending order of age are reported on the x-axis, individual antibody level for each assay/antigen is reported on the y-axis. Dashed lines indicate cut-off values as defined by Hicks et al. (10). The solid red line indicates linear least squares regression line and gray area indicates the 95% confidence interval.
