# Supplementary material for: Correlation between microneutralization test and a multiplexed immunoassay for evaluation of monkeypox and vaccinia virus antibodies before and after smallpox vaccination
Source: Front Immunol. 2025 Jun 23;16:1585284. doi: 10.3389/fimmu.2025.1585284 (PMC12229994; doi:10.3389/fimmu.2025.1585284)
Supplement: Supplementary file 1 [file Table1.docx]

| **Birth cohort** | **Antigen** | **Exposure group** | **N** | **Median** | **P25** | **P75** |
| --- | --- | --- | --- | --- | --- | --- |
| All | MXPV A29L | V+M+ | 101 | 2,421 | 731 | 6,413 |
| Born after 1979 | MXPV A29L | V+M+ | 15 | 452 | 329 | 827 |
| Born before 1975 | MXPV A29L | V+M+ | 86 | 3,859 | 1,083 | 7,026 |
| All | MXPV A29L | V+M- | 128 | 1,249 | 465 | 4,780 |
| Born after 1979 | MXPV A29L | V+M- | 44 | 599 | 172 | 1,997 |
| Born before 1975 | MXPV A29L | V+M- | 84 | 1,642 | 641 | 7,031 |
| All | MXPV A29L | V-M+ | 40 | 1,344 | 257 | 4,270 |
| Born after 1979 | MXPV A29L | V-M+ | 19 | 245 | 193 | 834 |
| Born before 1975 | MXPV A29L | V-M+ | 21 | 2,500 | 1,493 | 12,689 |
| All | MXPV A29L | V-M- | 100 | 526 | 211 | 1,472 |
| Born after 1979 | MXPV A29L | V-M- | 50 | 426 | 191 | 1,230 |
| Born before 1975 | MXPV A29L | V-M- | 50 | 685 | 272 | 1,664 |
| All | MXPV A35R | V+M+ | 101 | 17,308 | 5,058 | 47,986 |
| Born after 1979 | MXPV A35R | V+M+ | 15 | 582 | 201 | 12,931 |
| Born before 1975 | MXPV A35R | V+M+ | 86 | 19,795 | 6,372 | 52,485 |
| All | MXPV A35R | V+M- | 128 | 3,958 | 405 | 29,045 |
| Born after 1979 | MXPV A35R | V+M- | 44 | 237 | 164 | 926 |
| Born before 1975 | MXPV A35R | V+M- | 84 | 11,063 | 3,422 | 42,685 |
| All | MXPV A35R | V-M+ | 40 | 6,635 | 319 | 28,501 |
| Born after 1979 | MXPV A35R | V-M+ | 19 | 316 | 261 | 593 |
| Born before 1975 | MXPV A35R | V-M+ | 21 | 16,907 | 6,876 | 38,772 |
| All | MXPV A35R | V-M- | 100 | 828 | 174 | 4,827 |
| Born after 1979 | MXPV A35R | V-M- | 50 | 240 | 139 | 1,065 |
| Born before 1975 | MXPV A35R | V-M- | 50 | 1,955 | 682 | 12,702 |
| All | MXPV B6R | V+M+ | 101 | 17,521 | 3,024 | 46,034 |
| Born after 1979 | MXPV B6R | V+M+ | 15 | 587 | 186 | 8,808 |
| Born before 1975 | MXPV B6R | V+M+ | 86 | 20,780 | 4,516 | 53,952 |
| All | MXPV B6R | V+M- | 128 | 3,208 | 434 | 13,185 |
| Born after 1979 | MXPV B6R | V+M- | 44 | 221 | 120 | 977 |
| Born before 1975 | MXPV B6R | V+M- | 84 | 7,695 | 2,577 | 24,722 |
| All | MXPV B6R | V-M+ | 40 | 2,278 | 223 | 14,403 |
| Born after 1979 | MXPV B6R | V-M+ | 19 | 202 | 158 | 329 |
| Born before 1975 | MXPV B6R | V-M+ | 21 | 13,363 | 4,032 | 28,847 |
| All | MXPV B6R | V-M- | 100 | 685 | 154 | 4,649 |
| Born after 1979 | MXPV B6R | V-M- | 50 | 327 | 110 | 959 |
| Born before 1975 | MXPV B6R | V-M- | 50 | 2,324 | 303 | 9,437 |
| All | MXPV E8L | V+M+ | 100 | 5,530 | 1,647 | 15,668 |
| Born after 1979 | MXPV E8L | V+M+ | 15 | 610 | 184 | 2,866 |
| Born before 1975 | MXPV E8L | V+M+ | 85 | 6,362 | 2,547 | 18,953 |
| All | MXPV E8L | V+M- | 128 | 1,215 | 257 | 4,656 |
| Born after 1979 | MXPV E8L | V+M- | 44 | 222 | 156 | 851 |
| Born before 1975 | MXPV E8L | V+M- | 84 | 2,236 | 976 | 6,006 |
| All | MXPV E8L | V-M+ | 40 | 1,711 | 282 | 6,077 |
| Born after 1979 | MXPV E8L | V-M+ | 19 | 239 | 191 | 420 |
| Born before 1975 | MXPV E8L | V-M+ | 21 | 5,439 | 2,987 | 12,702 |
| All | MXPV E8L | V-M- | 100 | 779 | 182 | 1,880 |
| Born after 1979 | MXPV E8L | V-M- | 50 | 267 | 141 | 1,453 |
| Born before 1975 | MXPV E8L | V-M- | 50 | 1,045 | 394 | 3,271 |
| All | MXPV M1R | V+M+ | 101 | 1,625 | 612 | 6,749 |
| Born after 1979 | MXPV M1R | V+M+ | 15 | 624 | 263 | 1,436 |
| Born before 1975 | MXPV M1R | V+M+ | 86 | 2,029 | 713 | 8,665 |
| All | MXPV M1R | V+M- | 128 | 624 | 320 | 1,677 |
| Born after 1979 | MXPV M1R | V+M- | 44 | 355 | 219 | 600 |
| Born before 1975 | MXPV M1R | V+M- | 84 | 990 | 439 | 2,118 |
| All | MXPV M1R | V-M+ | 40 | 626 | 369 | 1,758 |
| Born after 1979 | MXPV M1R | V-M+ | 19 | 376 | 309 | 484 |
| Born before 1975 | MXPV M1R | V-M+ | 21 | 1,736 | 902 | 2,843 |
| All | MXPV M1R | V-M- | 100 | 329 | 214 | 956 |
| Born after 1979 | MXPV M1R | V-M- | 50 | 329 | 214 | 572 |
| Born before 1975 | MXPV M1R | V-M- | 50 | 355 | 215 | 1,005 |
| All | VACV A27L | V+M+ | 101 | 3,008 | 590 | 8,052 |
| Born after 1979 | VACV A27L | V+M+ | 15 | 388 | 281 | 657 |
| Born before 1975 | VACV A27L | V+M+ | 86 | 4,350 | 1070 | 8,879 |
| All | VACV A27L | V+M- | 128 | 1,433 | 513 | 5,324 |
| Born after 1979 | VACV A27L | V+M- | 44 | 871 | 373 | 2,374 |
| Born before 1975 | VACV A27L | V+M- | 84 | 1,906 | 727 | 7,802 |
| All | VACV A27L | V-M+ | 40 | 987 | 286 | 5,189 |
| Born after 1979 | VACV A27L | V-M+ | 19 | 271 | 193 | 524 |
| Born before 1975 | VACV A27L | V-M+ | 21 | 2,560 | 1007 | 9,620 |
| All | VACV A27L | V-M- | 100 | 506 | 262 | 1,503 |
| Born after 1979 | VACV A27L | V-M- | 50 | 410 | 221 | 1,526 |
| Born before 1975 | VACV A27L | V-M- | 50 | 607 | 303 | 1,488 |
| All | VACV A33R | V+M+ | 101 | 18,243 | 5,346 | 58,341 |
| Born after 1979 | VACV A33R | V+M+ | 15 | 533 | 166 | 15,001 |
| Born before 1975 | VACV A33R | V+M+ | 86 | 23,633 | 7,160 | 62,368 |
| All | VACV A33R | V+M- | 128 | 4,674 | 293 | 36,038 |
| Born after 1979 | VACV A33R | V+M- | 44 | 201 | 127 | 763 |
| Born before 1975 | VACV A33R | V+M- | 84 | 13,074 | 4,536 | 48,012 |
| All | VACV A33R | V-M+ | 40 | 7,145 | 290 | 37,052 |
| Born after 1979 | VACV A33R | V-M+ | 19 | 286 | 202 | 451 |
| Born before 1975 | VACV A33R | V-M+ | 21 | 27,086 | 10,949 | 81,695 |
| All | VACV A33R | V-M- | 100 | 850 | 175 | 6,749 |
| Born after 1979 | VACV A33R | V-M- | 50 | 219 | 120 | 1,109 |
| Born before 1975 | VACV A33R | V-M- | 50 | 2,513 | 521 | 11,607 |
| All | VACV B5R | V+M+ | 101 | 17,459 | 3,166 | 45,180 |
| Born after 1979 | VACV B5R | V+M+ | 15 | 868 | 197 | 9,233 |
| Born before 1975 | VACV B5R | V+M+ | 86 | 22,333 | 4,461 | 58,064 |
| All | VACV B5R | V+M- | 128 | 3,790 | 429 | 15,208 |
| Born after 1979 | VACV B5R | V+M- | 44 | 247 | 133 | 762 |
| Born before 1975 | VACV B5R | V+M- | 84 | 7,752 | 2,697 | 28,968 |
| All | VACV B5R | V-M+ | 40 | 2,008 | 242 | 18,324 |
| Born after 1979 | VACV B5R | V-M+ | 19 | 225 | 157 | 348 |
| Born before 1975 | VACV B5R | V-M+ | 21 | 17,459 | 4,365 | 34,539 |
| All | VACV B5R | V-M- | 100 | 694 | 160 | 4,889 |
| Born after 1979 | VACV B5R | V-M- | 50 | 337 | 130 | 1,093 |
| Born before 1975 | VACV B5R | V-M- | 50 | 2,515 | 268 | 12,047 |
| All | VACV D8L | V+M+ | 101 | 11,548 | 2,747 | 33,859 |
| Born after 1979 | VACV D8L | V+M+ | 15 | 450 | 256 | 5,188 |
| Born before 1975 | VACV D8L | V+M+ | 86 | 15,866 | 5,284 | 39,031 |
| All | VACV D8L | V+M- | 128 | 1,566 | 282 | 6,438 |
| Born after 1979 | VACV D8L | V+M- | 44 | 271 | 132 | 733 |
| Born before 1975 | VACV D8L | V+M- | 84 | 3,425 | 1,237 | 9,361 |
| All | VACV D8L | V-M+ | 40 | 3,446 | 309 | 11,476 |
| Born after 1979 | VACV D8L | V-M+ | 19 | 283 | 223 | 511 |
| Born before 1975 | VACV D8L | V-M+ | 21 | 10,871 | 5,458 | 35,695 |
| All | VACV D8L | V-M- | 100 | 563 | 147 | 2,419 |
| Born after 1979 | VACV D8L | V-M- | 50 | 217 | 140 | 993 |
| Born before 1975 | VACV D8L | V-M- | 50 | 1,251 | 248 | 5,478 |
| All | VACV L1R | V+M+ | 101 | 1,988 | 660 | 7,684 |
| Born after 1979 | VACV L1R | V+M+ | 15 | 647 | 306 | 1,711 |
| Born before 1975 | VACV L1R | V+M+ | 86 | 2,513 | 904 | 8,921 |
| All | VACV L1R | V+M- | 128 | 833 | 350 | 1,983 |
| Born after 1979 | VACV L1R | V+M- | 44 | 378 | 228 | 741 |
| Born before 1975 | VACV L1R | V+M- | 84 | 1,214 | 512 | 2,975 |
| All | VACV L1R | V-M+ | 40 | 711 | 377 | 2,295 |
| Born after 1979 | VACV L1R | V-M+ | 19 | 387 | 320 | 494 |
| Born before 1975 | VACV L1R | V-M+ | 21 | 2,164 | 1,099 | 3,136 |
| All | VACV L1R | V-M- | 100 | 443 | 239 | 1,108 |
| Born after 1979 | VACV L1R | V-M- | 50 | 401 | 228 | 677 |
| Born before 1975 | VACV L1R | V-M- | 50 | 528 | 296 | 1,274 |

**Table 1S.** Median antibody levels and interquartile range (25^th^ percentile, P25 and 75^th^ percentile, P75) for each antigen by exposure group (VACV+ MPXV+, V+M+; VACV+ MPXV-, V+M-; VACV- MPXV+, V-M+; VACV- MPXV-, V-M-) and birth cohort. VACV, Vaccinia virus; MPXV, Monkeypox virus).
