# Supplementary figures and images for: Correlation between microneutralization test and a multiplexed immunoassay for evaluation of monkeypox and vaccinia virus antibodies before and after smallpox vaccination
Source: Front Immunol. 2025 Jun 23;16:1585284. doi: 10.3389/fimmu.2025.1585284 (PMC12229994; doi:10.3389/fimmu.2025.1585284)

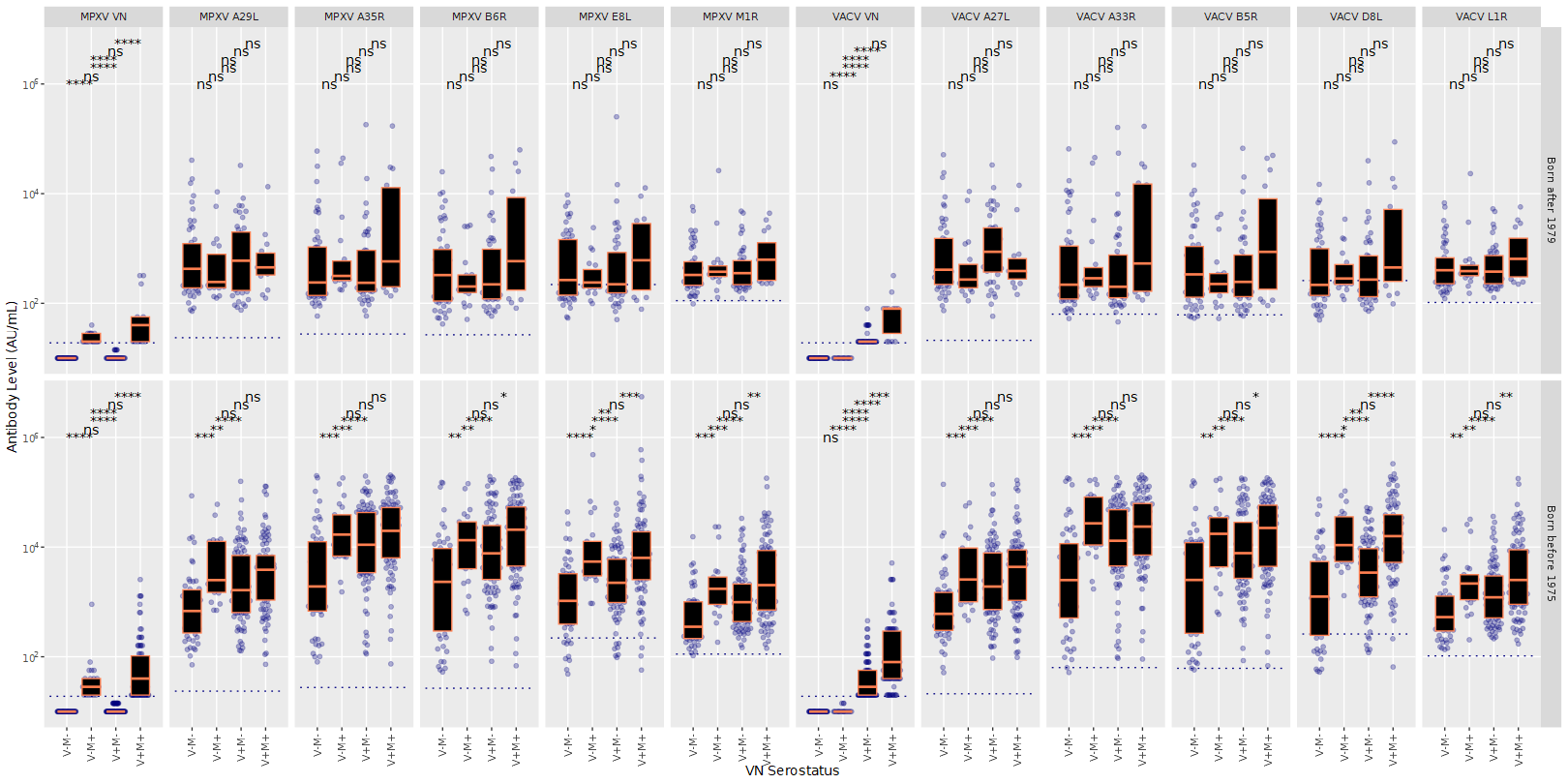

Supplement: Supplementary Figure 1 — Antibody levels (AU/ml) per monkeypox (MPXV, left panels) and vaccinia (VACV, right panels) antigens per birth cohort (born after 1979, upper panels; born after 1975, lower panels). Exposure groups (VN serostatus) are reported on the x-axis, antibody levels for each subject are reported on the y-axis. Boxes represent median and interquartile range. Dashed lines indicate cut-off values as defined by Hicks et al. (10). Significances from Kruskal-Wallis test followed by Dunn test are indicated: ns, not significant, *P < 0.05, **P < 0.01, ***P < 0.001. ****P < 0.0001. [file Image1.tif]

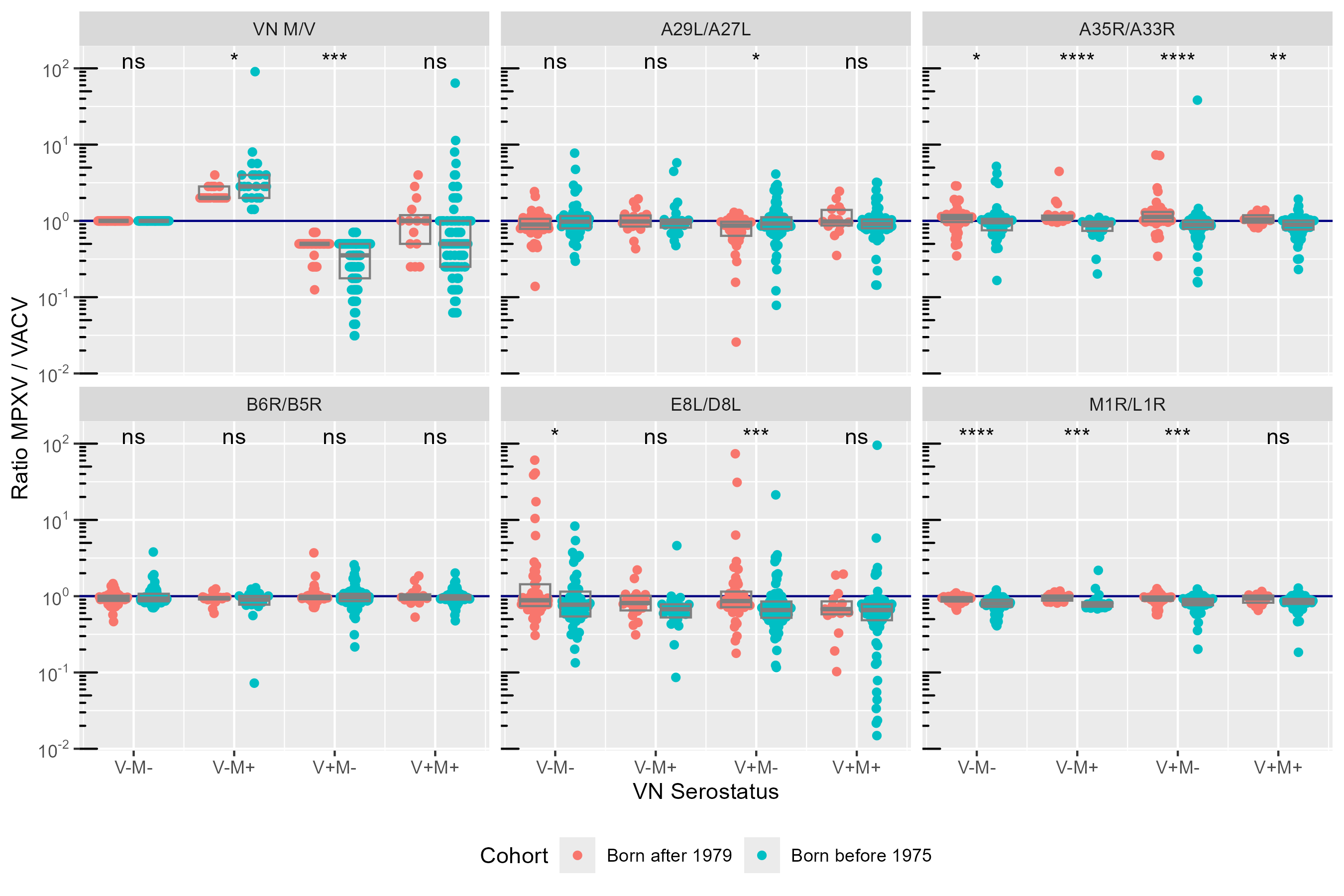

Supplement: Supplementary Figure 2 — Ratio Monkeypox (MPXV)/Vaccinia (VACV) for each ortholog pair by exposure group and birth cohort. Exposure groups (VN serostatus) are reported on the x-axis, MPXV/VACV ratio for each subject is reported on the y-axis. Colors indicate birth cohort (red: born after 1979; blue: born before 1975). Boxes represent median and interquartile range. Significances from Mann-Whitney U test are indicated: ns, not significant, *P < 0.05, **P < 0.01, ***P < 0.001. ****P < 0.0001. [file Image2.tif]

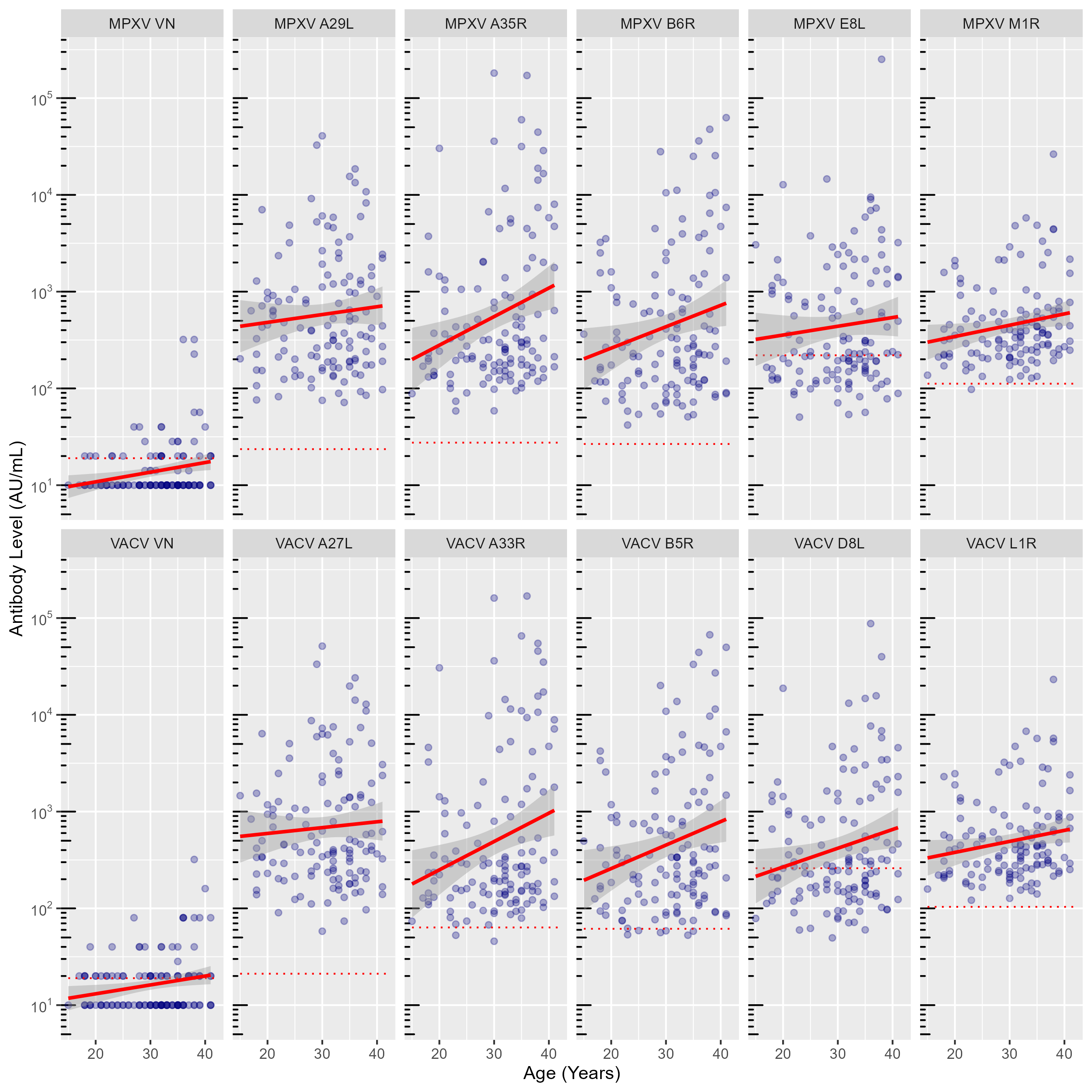

Supplement: Supplementary Figure 3 — Correlation between antibody levels and age for the born after 1979 cohort. The subjects in ascending order of age are reported on the x-axis, individual antibody level for each assay/antigen is reported on the y-axis. Dashed lines indicate cut-off values as defined by Hicks et al. (10). The solid red line indicates linear least squares regression line and gray area indicates the 95% confidence interval. [file Image3.tif]

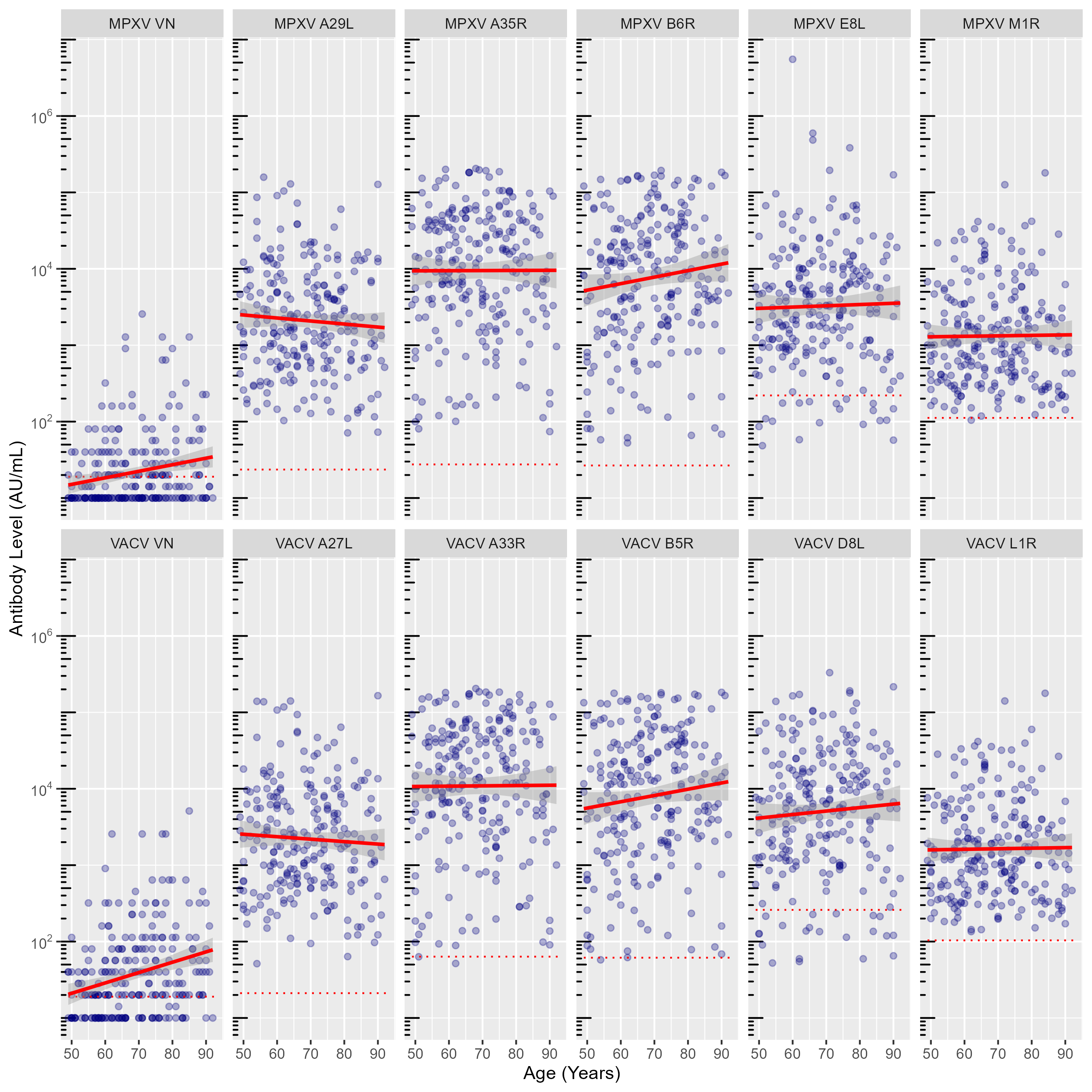

Supplement: Supplementary Figure 4 — Correlation between antibody levels and age for the born before 1975 cohort. The subjects in ascending order of age are reported on the x-axis, individual antibody level for each assay/antigen is reported on the y-axis. Dashed lines indicate cut-off values as defined by Hicks et al. (10). The solid red line indicates linear least squares regression line and gray area indicates the 95% confidence interval. [file Image4.tif]
